# TISSUE EXPRESION OF THE GENES MUTYH AND OGG1 IN PATIENTS WITH SPORADIC COLORECTAL CANCER

**DOI:** 10.1590/0102-6720201700020005

**Published:** 2017

**Authors:** Enzo Fabrício Ribeiro NASCIMENTO, Marcelo Lima RIBEIRO, Daniela Oliveira MAGRO, Juliana CARVALHO, Danilo Toshio KANNO, Carlos Augusto Real MARTINEZ, Cláudio Saddy Rodrigues COY

**Affiliations:** 1 Faculty of Medical Sciences of the State University of Campinas (FCM-UNICAMP), Postgraduate Program in Surgery Sciences, Campinas, SP;; 2 São Francisco University, Post-Graduation Program in Health Sciences, Bragança Paulista, SP;; 3 Faculty of Medical Sciences of the State University of Campinas, Department of Surgery, Campinas, SP;; 4 State University of Campinas, Integrated Center for Women’s Health Care, Campinas, SP, Brazil.

**Keywords:** Colorectal cancer, Genes, DNA repair, Oxidative stress, DNA Glycosolasys.

## Abstract

**Background:**

MTUYH and OGG1 genes have importance in the base excision repair systems of oxidized DNA bases. Modification of the tissue expression of these genes is related to the increased risk of developing colorectal cancer.

**Aim:**

To evaluate the tissue expression of MUTYH and OGG1 comparing normal and neoplastic tissues of patients with sporadic colorectal cancer and to correlate it with clinical and histopathological variables.

**Method:**

MUTYH and OGG1 tissue expression was quantified by RT-PCR in patients with colorectal cancer and the values were compared in normal and neoplastic tissues. MUTYH and OGG1 expression was measured and normalized to the constitutive 18S gene. The level of expression of both genes was correlated with the variables: age, gender, tumor location, size of the tumor, histological type, degree of cell differentiation, invasion depth in the intestinal wall, angiolymphatic infiltration, lymph node involvement and TNM staging.

**Results:**

Was found downregulation of both genes in neoplastic when compared to normal tissue. There was downregulation of the MUTYH in larger tumors and in patients with angiolymphatic invasion. Tumors with more advanced TNM stages (III and IV) presented downregulation of both genes when compared to those with earlier stages (I and II).

**Conclusion:**

The MUTYH and OGG1 genes present downregulation in the more advanced stages of colorectal cancer.

## RESUMO

**Racional:**

Os genes MUTYH e OGG1 possuem importância nos sistemas de reparo por excisão de bases oxidadas do DNA. Modificação na expressão tecidual desses genes encontra-se relacionada ao maior risco do desenvolvimento do câncer colorretal. ***Objetivo***: Avaliar a expressão tecidual dos genes MUTYH e OGG1 comparando tecidos normais e neoplásicos de portadores de câncer colorretal esporádico e correlacioná-la com variáveis clínicas e histopatológicas.

**Método:**

Avaliou-se por PCR, em tempo real, a expressão tecidual dos genes MUTYH e OGG1 em 49 portadores de câncer colorretal comparando tecidos normais e neoplásicos. A expressão dos genes MUTYH e OGG1 foi quantificada e normalizada com o gene constitutivo 18S. A intensidade de expressão de ambos os genes foi correlacionada as variáveis: idade, gênero, localização do tumor, tamanho do tumor, tipo histológico, grau de diferenciação celular, profundidade de invasão na parede intestinal, invasão angiolinfática, linfonodos comprometidos e estadiamento TNM.

**Resultados:**

Encontrou-se menor expressão de ambos os genes no tecido neoplásico quando comparado ao tecido normal. Houve menor expressão do gene MUTYH nos tumores com maiores dimensões e nos doentes que apresentavam invasão angiolinfática. Tumores com estadios mais avançados (III e IV) apresentavam expressão menor de ambos os genes quando comparados àqueles com estadios mais precoces (I e II).

**Conclusão:**

Os genes MUTYH e OGG1 apresentam menor expressão tecidual nos estadios mais avançados do câncer colorretal.

## INTRODUCTION

According to recent epidemiological inquiry, colorectal cancer (CRC) occurred in 2015, 1.4 million people worldwide being the third type of most common malignant neoplasm among men and the second among women^9^. The National Institute of Cancer (INCA) estimates that for the biennium 2016-2017 In Brazil, depending on the geographical region considered, the CRC can affect of 3.35 to 28,15/100,000 inhabitants among men and 2.09 to 29,13/100,000 inhabitants among women^13^. With the increase in life expectancy, the phenomena of globalisation at greater exposure to carcinogenic agents and, especially, the change of dietary habits it is expected that the CRC has increasing importance in the profile of mortality from cancer throughout the world considerably increasing the economic and social costs^19^ ^,^ ^23^. 

Genetic studies have shown development of sporadic CRC is an evolutionary process that involves a series of sequential mutations or modifications in the expression of genes related to cell cycle^21^. It is already well established that the development of the CRC from the normal mucosa, is mediated by a sequence of mutations of genes controllers of cell cycle (the proliferation, differentiation, apoptosis and DNA repair)^8^ ^,^ ^30^. Mutations in these genes can confer additional advantages for growth of tumor tissue in relation to normal tissue^19^. 

Although the initial phenomena of colorectal carcinogenesis are still a reason for constant research, it has been demonstrated that DNA damage caused by reactive oxygen species (ROS) is related to the development of CRC^34^ ^,^ ^27^ ^,^ ^31^ ^,^ ^20^ ^,^ ^4^. The ROS are produced in large quantities in chronic inflammatory processes affecting the intestinal epithelium. These radicals induce persistent damage to DNA and, if they are not readily inhibited by antioxidant systems can cause mutations related to the development of the CRC^16^. One of the mechanisms of DNA damage to more well-studied relates to the oxidation of the base fertilization guanine, forming the 8-oxoguanina (8-oxoG). The 8-oxoG is a highly oxidized mutagenic capable of causing transversions of CC→TA tapes of DNA allowing, if not repaired, the appearance of mutations. When these mutations compromise tumor suppressor genes or oncogenes may arise a clone of cells with proliferative autonomy and uncontrolled growth, changes inherent to the neoplastic cells^31^.

It is already well established that the oxidation of bases of DNA allows for the development of the CRC and that removal of these databases oxidized is vital to avoid the appearance of mutations^26^. A chronic imbalance between the mechanisms of damage and DNA repair increases the risk of genomic instability and, therefore, it is important to understand the mechanisms by which the repair systems that remove the oxidized bases of DNA present in the genome. For both, living organisms have specific genes to correct the errors of matching caused by oxidative stress. The system of excision of bases (BER) is the primary way to repair DNA responsible for correction of oxidized bases, having a fundamental role in preserving the integrity of the DNA submitted to the deleterious action of ROS^28^ ^,^ ^24^. Among all the genes involved in repairing and removing the 8-oxoG, genes MUTHY and OGG1, components of the system BER, have a prominent role. These genes encode DNA-glicosilases which has the function of removing the oxidized bases paired erroneously, ensuring the maintenance of the integrity of the tapes of DNA. Recent studies suggest that patients with CRC have lower expression of genes OGG1 and mutyh in tissue cancer when compared to normal tissues suggesting that these systems operate without a disability^33^. However, the correlation between the level of tissue expression and clinical variables and histopathological were still little studied^33^. The objective of this study was to evaluate the tissue expression of genes MUTYH and OGG1 in normal tissue and neoplastic patients with CRC sporadic and correlate it to the main clinical and histopathological.

## METHOD

This study was submitted to and approved by the Research Ethics Committee at the Universidade São Francisco in Bragança Paulista (Project No. 0235.0.142.000-07). All patients who have provided biological material for the present study signed an informed consent form agreeing to participate in all stages, and were informed of the purpose of the research.

### Casuistry

Were studied prospectively 49 patients being 26 (53%) women with an average age of 65.8±11.3 years, suffering from adenocarcinoma of the colon and rectum, surgery with curative intent. Were excluded by means of criteria of clinical and endoscopic patients suspected of hereditary CRC (adenomatous polyposis familial syndrome and Lynch), sick undergoing treatment neoadjuvant chemoradiation therapy, patients with CRC associated with inflammatory bowel disease, less than 18 years, sick submitted to surgical treatment in urgent circumstances, and those who refused to participate in the study. The patients selected for the study were submitted to clinical staging, laboratory and imaging examinations in accordance with the guidelines recommended by the American Society of Colorectal Surgeons^5^. 

### Sample collection 

Immediately after the removal of the surgical specimen were collected fragments of tissue obtained from the mucosa normal colic (at least 10 cm from the proximal margin of the lesion), and the periphery of the neoplastic lesion. After collection, the fragments were identified with the record of the patient name, date and place of where they had been collected. The fragments were placed in tubes suitable for storage in ultra-cooling and immediately stored at -80° C until the time of processing. The data, epidemiological, clinical and pathological findings were obtained from medical records. Histopathological data such as location of the tumor, macroscopic aspect of the tumor, lesion size, histological type, degree of cellular differentiation, depth of invasion in the intestinal wall, presence of lymphatic invasion or perineural, number of lymph nodes resected, number of lymph nodes involved, reason of lymph nodes involved and staging TNM were extracted from the histopathological report prepared by a single pathologist with expertise in colorectal neoplasia. The staging of tumors followed the TNM Classification according to the 6th edition proposed by the UICC (International Union of Cancer Control). All the sick were treated at the outpatient clinic of a colorectal neoplasia of Hospital Universitário São Francisco, Bragança Paulista, SP, Brazil, by the same professional.

### Extraction of RNA and RT-PCR (Real-time polymerase chain reaction)

Total RNA was isolated using the kit of tissue RNeasy (Qiagen). The purity was assessed using the NanoDrop 2000 spectrophotometer (Thermo Scientific, Wilmington, DE, USA). The single strand of cDNA was synthesized from RNA using the kit for high capacity storage of cDNA (Applied Biosystems, Foster City, CA, USA) following the manufacturer’s protocol. 

Quantitative PCR was performed using a system of 7500 real time PCR (Applied Biosystems) using the numbers of cycles limit determined by the software RQ Study (Applied Biosystems). The reactions were racing in triplicate, and the numbers of cycle threshold were considered by the average. The mixture of the reaction containing a total of e 50µl It was prepared as follows: 25 µl SYBR Green™, PCR SuperMix UDG (Invitrogen Life Technologies, Alemeda, CA, EUA), 10 mM for each primer (Table 1), e 1 µl de cDNA (100 ng). The first cycle was performed with a preliminary treatment with UDG for 2 min at 50° C and denaturation by more 2 min to 95° C, followed by 45 cycles of denaturation at 95° C for 15s, annealing at 60º C for 15 s and extension of the primer at 72º C during 15 s. This step was followed by an analysis of melting points of amplification products of dual tape consisting of 40 cycles of 1° C decreased (15 s each) starting at 95° C. The first derivative of this map, dF/dT, represents the rate of change of fluorescence in the reaction. A significant change in the fluorescence occurs at the point of fusion of free dual chain found in the PCR. A map of dF/dT as a function of temperature shows these changes as distinct peaks.

**TABLE 1:** Primers used for RT-PCR (attachment)

Gene	Sequence (5’-3’)
18S	CGCGGTTCTATTTTGTTGGT CGGTCCAAGAATTTCACCTC
MUTYH	CCTGTGGGGACCTTATGCT CCTTTGGAACCCTTTCTGC
OGG1	CCTGTGGGGACCTTATGCT CCTTTGGAACCCTTTCTGC

The expression of genes MUTYH and OGG1 expression was measured and normalized for the gene incorporation 18S, which showed a constant expression in all samples tested. The expression on was calculated according to the formula 2 ^-∆∆Ct^. And the results were expressed as mean of expression of the gene ±EP

### Statistical analysis

Were performed using statistical software version 13.0 SPSS (SPSS, Chicago, IL). The values found for the expression of the genes in the normal tissues and in the considered variables were identified by descriptive statistics and expressed as the average expression of the gene with its respective standard error. The comparison of the gene expression between the normal and neoscopic tissues, as well as between the proposed variables was performed using the parametric Mann-Whitney test. Values of p≤0.05 were significant values obtained in the comparison between normal and neoplastic tissues were identified with a cross (†), while the values obtained comparing the stratifications between the clinical and histopathological variables with an asterisk (*).

## RESULTS

### Population of patients

Table 2 shows the distribution of the sample in relation to the variables considered in the study.

**TABLE 2:** Relationship of the variables studied

Characteristics	Number of Patients (%)
Genre Male Female	23/49 (47%) 26/49 (53%)
Age 41-51 years 52-62 years >63 years	5/49 (10%) 9/49 (19%) 35/49 (71%)
Location Colon Rectum	17/49 (35%) 32/49 (65%)
**Tumor size** ≤ 3 cm > 3 cm	14/30 (47%) 16/30 (53%)
**Histological type** Usual Mucoprodutor	47/49 (96%) 2/49 (4%)
**Degree of cell differentiation** Good Moderately Little	5/49 (10%) 38/40 (78%) 6/49 (12%)
**Degree of invasion of the intestinal wall** T1 - T2 T3 -T4	38/49 (78%) 11/49 (22%)
**Resected lymph nodes** ≤12 >12	27/49 (55%) 32/49(65%)
**compromised lymph nodes** N0 N1-N2	29/49 (59%) 20/49 (41%)
**Reason for compromised lymph nodes** 0 - ≤ 10% > 11% - ≤ 20% > 21%	24/49 (49%) 7/49 (14%) 18/49 (37%)
**Angiolymphatic invasion** Yes No	17/49 (35%) 32/49 (65%)
**Perineural Invasion** Yes No	27/49 (55%) 22/49 (45%)
Staging TNM I and II III and IV	19/49 (39%) 30/49 (61%)

Table 3 shows the expression of genes MUTYH and OGG1 with respect to the characteristics of the studied samples, as well as between the normal tissue and neoplastic, considering only the variables where there was a statistically significant difference in one of the genes.

**TABLE 3:** Gene expression OGG1 and MUTH according to the characteristics of the sample

Characteristic OGG1		Gene	
		MUTYH	
Fold induction	Tumor/Normal	0.28 ± 0.12†	0.41±0.02†
Location	Cólon Reto	1.09 ± 0.38 0.48 ± 0.02*	0.69 ± 0.34 0.50 ± 0.10
Tumor size	≤ 3cm > 3cm	0.21±0.05 0.22±0.05	0.36±0.08* 0.13±0.02
Angiolymphatic invasion	Sim Não	0.24 ± 0.04 0.29 ± 0.09	0.30 ± 0.09* 0.89 ± 0.11
TNM (Stadium)	I - II III - IV	0.49 ± 0.12 0.16 ± 0.08*	0.78 ± 0.20 0.33 ± 0.07*

† = p < 0.05 When compared to normal tissue; ** p < 0.05 When comparing the characteristics

## DISCUSSION

The human genome is particularly vulnerable to oxidative stress. The ROS in the cells of the mucosa colic are formed as products of the normal aerobic metabolism or as a result of exposure of the cells to ionizing radiation, chemicals, ischemia, inflammation acute and chronic or, even, for the simple modification in the supply of energy substrate to normal colonocites represented by short-chain fatty acids^20, 12^. As they are electrophilic molecules with the ability highly reactive, ROS attack substances with high electron density such as the nitrogenous bases that make up DNA^3^. These radicals can cause breakage single or double quote in the tapes of the DNA or oxidize their nitrogenous bases allowing for the development of errors of matching and, consequently, the development of mutations^7^ ^,^ ^2^. When we consider the environmental factors related to the development of the CRC, studies have shown that oxidative stress is one of the main mechanisms responsible for the appearance of mutations related to the development of the disease^6^ ^,^ ^2^. It has been demonstrated that the chronic inflammation and continuously in the intestinal mucosa, as occurs in the intestinal inflammatory diseases, increasing the production of ROS, increases the possibility of developing of CRC^31^. Further strengthens this possibility the results of studies showing that the levels of oxidative stress in the neoplastic tissue of patients with CRC is significantly higher when compared to the normal tissue^26^ ^,^ ^27^. 

The oxidative damage most often caused in the DNA by exposure to the RLO is the formation of 8-oxoG. The neoplastic tissue of patients with CRC has on average two times more 8-oxoG when compared to normal mucous^17.^

In order to correct the errors of matching caused by 8-oxoG and prevent the emergence of mutations, the body has different systems of DNA repair. The system BER is the main responsible for excision of oxidized bases^22^. The genes MUTYH, OGG1, MTH1, NEIL1, APEX1, PARP1, LIG3, MGP e NTHL1 are the main components of the system BER. 

The genes MUTYH It is located on the short arm of chromosome 1 (1p32-34) and has 16 exons. Encodes a DNA-ung formed by 535 amino acids with a molecular weight of 39 kD, called MUTHY ung that operates in the DNA-glycosylase repair by excision of bases. During DNA duplication, the adenine base was usually matched to thymine (A-T), while guanine matched with the cytosine (C-G). During normal cellular aerobic metabolism or in situations that cause the increase of ROS production, the oxidation of guanine can occur to 8-oxoG. This oxidized base matched quickly and erroneously with adenine instead of cytosine. MUTY-glycosylase acts by removing from the DNA strand adenine bases poorly coupled with 8-oxoG. The enzyme corrects this error so that the development of mutations by type G:C>T:A which can lead to tumor formation if they compromise cell cycle controlling genes. The importance of mutations in the MUTYH gene in colorectal carcinogenesis was highlighted by the Al-Tassan et al. In 2002, where the authors demonstrated for the first time, individuals from the same family who presented biallelic germline mutations in the MUTYH gene and who had recessive forms of familial adenomatous polyposis (FAP), associated with the development of colorectal adenomas presenting progression to CRC in age when compared to the classic forms of FAP^1^. When analyzing the neoplastic tissues of these patients they found a high index of transversions of the type G:C>T:A in commonly mutated genes in the CRC (APC e K-RAS). Later, other authors confirmed these findings showing that mutations in the MUTYH gene, reducing the effectiveness of the BER system, predisposes to the appearance of CRC^1^ ^,^ ^14^ ^,^ ^15^.

The OGG1 gene is located on the short arm of the chromosome 3 (3p25.3). It consists of 12 exons and encodes the enzyme 8-oxoguanine glycosylase. It is estimated that the enzyme contains 424 amino acids in its molecule and molecular weight of 47kD^25^. It was demonstrated that the gene OGG1 has an essential role in repairing BER correcting transversions G:C>T:A. The enzyme transcribed, promotes the hydroxylation of connections glicofílicas formed between the 8-oxoG and the fraction of sugar from the basis of fertilization, removing the molecule of 8-oxoG and forming a site apurinic on tape of DNA that after the excision is subsequently filled with the correct fertilization. By its fundamental role in the system BER, mutation and polymorphisms in the gene OGG1 are considered events that increase the susceptibility to various forms of cancer. Studies have shown that reducing the activity of the gene could contribute to the development of CRC^32^.

A considerable number of hereditary CRC shows genetic or epigenetic genes defects on the system repair. The possibility that also occur changes in the system BER in the CRC sporadically is a plausible hypothesis. So, the research profile of expression of genes of the system BER related to correction of oxidized bases comparing normal tissue and neoplastic, can improve the understanding of the role of these genes in patients with sporadic CRC^32^. Previous studies have demonstrated that there is less efficiency of the system BER in patients with CRC when compared to healthy volunteers^15^ ^,^ ^33^. However, the mutations of the MUTYH gene and OGG1 in patients with sporadic CRC are not frequent and are influenced by the ethnic group studied^14^ ^,^ ^15^ ^,^ ^33^. Thus, in order to evaluate the mutations in these genes it is necessary to study a large contingent of patients belonging, if possible, the same ethnicity. A study evaluating the importance of mutations in the MUTYH to examine their prevalence in polish ethnicity found that among 1042 patients only 0.4% of the patients had a mutation biallelic associated with the development of adenomas and CRC^15^. Other authors by analyzing the contribution of mutations in germ MUTYH in the development of the CRC have evaluated 358 patients were not selected^10^. They found two patients (0.6%) with germ mutations biallelics and eight (2.2%) monoallelics mutations in the gene MUTYH. Patients with biallelic mutation had multiple adenomas, but not adenomatous polyposis profuse and, in both cases, the tumors were located in the distal colon. These results suggest that mutations biallelics of gene despite increasing the formation of adenomas and, consequently, the chance of developing cancer is likely to represent less than 3% of cases of sporadic CRC^10^. 

This study evaluated the expression of tissue several genes of the system BER and the ability to repair DNA of these genes by comparing normal tissue and neoplastic and correlating them with clinical variables and pathological^32^. The authors found that the expression of tissue gene OGG1 was significantly lower in tissues, neoplastic while the gene MUTYH showed no significant differences. They found that, to relate the expression of genes OGG1 and MUTYH when gender, age, location of the tumor histological grade stadiums of the TNM classification (I+II+x+II+IV) found no significant differences^32^. In Brazil, only one study evaluated the behavior of the gene OGG1 in patients with sporadic CRC^29^. Using samples from the same population of patients the authors found a lower expression of OGG1 in tumoral tissue^29^. With regard to location of the tumor identified lower gene expression only in patients with cancer of the rectum and in tumors with more advanced stages^29^.

In the present study we found similar results. When evaluating the expression of OGG1 we found reduced expression in tumor tissue, similar to the studies previously cited^32^ ^,^ ^29^. We also found a reduction in the tissue expression of the gene in patients with tumors located in the rectum and in patients classified in more advanced stages of the disease. With respect to expression of the gene MUTYH, despite being lower in neoplastic tissue, we found no statistically significant differences (p=0.06). It is possible that these values could present significant differences if they were included a larger number of patients. When you relate gene expression MUTYH with variables selected for the present study found that there was less expression of the gene in tumors that had diameter greater than 3 cm, in presenting angiolymphatic invasion and those classified in the more advanced stages (T3-T4) of the TNM classification. These findings suggest that the lower expression in these patients may contribute to the worse prognosis of the disease, since it is related to variables that confer a worse prognosis of the disease.

When we consider the results of the present study, and having science that there is a higher degree of oxidative stress in tissues neoplastic, it is possible that the lower expression of genes OGG1 and MUTYH in tumor tissue, as well as in the variables related to a worse prognosis of the disease, may occur due to the accumulation epigenetic mutations or hypermethylation in these genes by reducing their capacity to repair and favoring the development of a phenotype more aggressive as shown above^11^. The hypermethylation of the promoter region of genes that make up the system BER has been found in a variety of tumors (thyroid, bladder, ovaries, the brain as well as in the CRC)^18^.

Considering that genes OGG1, MUTYH, PARP-1 and XRCC1 are part of the same repair system (BER) it is possible that the presence of polymorphisms in these genes may interfere with their tissue expression. Recent study evaluating the influence of the APE1 T2197G polymorphism (asp148Glu) showed that this alteration interferes in the expression of the genes of the BER system when comparing normal and neoplastic tissues. The results found in the present study not only confirm previous results but also reinforce the importance of repair integrity by excision of bases in the etiopathogenesis and progression of sporadic CRC. 

## CONCLUSION

The MUTYH and OGG1 genes present downregulation in the more advanced stages of colorectal cancer.
